# Using modern plant trait relationships between observed and theoretical maximum stomatal conductance and vein density to examine patterns of plant macroevolution

**DOI:** 10.1111/nph.13579

**Published:** 2015-07-31

**Authors:** Jennifer C. McElwain, Charilaos Yiotis, Tracy Lawson

**Affiliations:** ^1^ Earth InstituteO'Brien Centre for ScienceUniversity College DublinBelfieldIreland; ^2^ School of Biology and Environmental ScienceUniversity College DublinBelfieldIreland; ^3^ School of Biological ScienceUniversity of EssexColchesterCO4 3SQUK

**Keywords:** evolution of angiosperms, functional traits, maximum theoretical stomatal conductance (*g*_max_), palaeophysiology, plasticity, stomatal density, stomatal evolution, vein density (*D*_v_)

## Abstract

- Understanding the drivers of geological‐scale patterns in plant macroevolution is limited by a hesitancy to use measurable traits of fossils to infer palaeoecophysiological function.
- Here, scaling relationships between morphological traits including maximum theoretical stomatal conductance (*g*_max_) and leaf vein density (*D*_v_) and physiological measurements including operational stomatal conductance (*g*_op_), saturated (*A*_sat_ *)* and maximum (*A*_max_) assimilation rates were investigated for 18 extant taxa in order to improve understanding of angiosperm diversification in the Cretaceous.
- Our study demonstrated significant relationships between *g*_op_, *g*_max_ and *D*_v_ that together can be used to estimate gas exchange and the photosynthetic capacities of fossils. We showed that acquisition of high *g*_max_ in angiosperms conferred a competitive advantage over gymnosperms by increasing the dynamic range (plasticity) of their gas exchange and expanding their ecophysiological niche space. We suggest that species with a high *g*_max_ (> 1400 mmol m^−2^ s^−1^) would have been capable of maintaining a high *A*_max_ as the atmospheric CO_2_ declined through the Cretaceous, whereas gymnosperms with a low *g*_max_ would experience severe photosynthetic penalty.
- Expansion of the ecophysiological niche space in angiosperms, afforded by coordinated evolution of high *g*_max_ *, D*_v_ and increased plasticity in *g*_op_ *,* adds further functional insights into the mechanisms driving angiosperm speciation.

## Introduction

Examination of large scale ecological, ecophysiological and physiognomic datasets in plant biology has revealed important trait relationships that are conserved across species (Wright *et al*., 2004; Grime, 2006; Reich *et al*., 2007; Kattge *et al*., 2011; Yang *et al*., 2015). These suites of correlated traits have enabled classification of extant taxa into broad ecological categories – such as plant functional types – which serve as the starting point for mapping and predicting vegetation responses to past and future global change. They have also contributed strongly to the development of palaeoclimate proxies (Yang *et al*., 2015). Ongoing studies of extant plant trait relationships are beginning to have a significant impact on the understanding of plant macroecological and macroevolutionary processes in the fossil plant record by providing critical insights into the palaeophysiology and general functional attributes of fossil taxa (Beerling & Woodward, 1997, 2001; Franks & Beerling, 2009a; Wilson & Knoll, 2010; de Boer *et al*., 2012; Lee *et al*., 2015) including those that are extinct (Wilson *et al*., 2008). Many trait based datasets incorporate only functional or only morphological/morphometric traits, yet integration of both data types (such as exemplified by TRY; Kattge *et al*., 2011) is required if any inferences on the palaeophysiology of fossil taxa from measured morphological attributes is to be made. Gaining insights on the functional biology of fossil taxa will permit a more nuanced assessment of plant macroevolutionary patterns from the fossil plant record.

A compelling example of how modern trait based datasets can be used to gain novel insights into the mechanisms driving plant macroevolution is the ‘vein density hypothesis’ of angiosperm evolution, which uses changes in Cretaceous fossil plant morphological traits (in this case leaf vein density) to reconstruct maximum conductive and photosynthetic capacity of angiosperms vs gymnosperms in Cretaceous fossil floras (Boyce *et al*., 2009; Brodribb & Feild, 2010; Feild *et al*., 2011b). According to this hypothesis angiosperms uniquely evolved the capacity to increase leaf vein density above *c*. 6 mm mm^−2^ in the mid Cretaceous *c*. 100 million yr ago (Mya; late Albian–early Cenomanian periods) (de Boer *et al*., 2012). This anatomical innovation enabled angiosperms to outcompete incumbent gymnosperms (with low vein densities) as it removed a developmental constraint on potential productivity. Supplying more water via a high vein density network to stomata enabled greater transpiration (Boyce *et al*., 2009; Brodribb & Feild, 2010) and ultimately enhanced the photosynthetic capacity (Brodribb *et al*., 2007; de Boer *et al*., 2012).

Another example of how the observed morphological traits measured in fossils are used to estimate palaeophysiology is demonstrated in the palaeo‐proxy CO_2_ model of Franks *et al*. (2014). This mechanistic model uses a scaling relationship between the maximum theoretical stomatal conductance (*g*_max_ in mmol m^−2^ s^−1^), calculated from the density (SD), size and geometry of stomata when fully open (Parlange & Waggoner, 1970; Franks & Beerling, 2009b) and measured conductance values (*g*_op_) to infer stomatal conductance of fossil taxa. It is widely known that because stomata respond dynamically with the environment, the anatomical *g*_max_ is rarely observed in field conditions (Lawson & Morison, 2004; Dow *et al*., 2014) and that the operational stomatal conductance of a leaf, which we refer to here as *g*_op_, is usually measured at much lower values than *g*_max_ (Franks *et al*., 2009, 2014). Quantification of the scaling relationship between *g*_op_ and *g*_max_ has, however, only been undertaken in detail for two extant angiosperm species – *Eucalyptus globulus* (Franks *et al*., 2009) and *Arabidopsis thaliana* (Dow *et al*., 2014) and it is not known whether a universal relationship exists across many species. This currently hampers a wider application to the fossil record and integration with other likely correlated functional traits such as vein density.

There is close developmental and physiological coordination of water supply via veins and water loss via stomata at the leaf level (Sack *et al*., 2003; McElwain, 2011; Brodribb *et al*., 2013) yet the role of stomatal evolution as a potential driver or accessory to vein density evolution has not been systematically investigated. Furthermore, there are no modern trait datasets that incorporate *D*_v_, *g*_max_, *g*_op_ and photosynthetic traits across species. The expectation is that the maximum stomatal conductance should follow a similar evolutionary trajectory in angiosperms and gymnosperms as vein density (Boyce *et al*., 2009). A primary objective of this study therefore was to investigate the role of stomatal evolution in the ecological and evolutionary success of angiosperms compared with gymnosperms by undertaking a comparative study on plasticity in *g*_op_ in relation to the theoretical maximum stomatal conductance (*g*_max_) and vein density (*D*_v_). Specifically we ask: is there a universal scaling relationship between *g*_op_ and *g*_max_; did an increase in maximum gas exchange capacity, facilitated by high *g*_max_ and *D*_v_, enable angiosperms to increase plasticity in their day‐to‐day operational range of stomatal conductance (*g*_op_) compared with gymnosperms; and what are the likely evolutionary implications of a greatly expanded *g*_op_ range in terms of ecological competition, resource use and assimilation rates in a high CO_2_ world of the Cretaceous (*c*. 2000 ppm) compared with that of today (*c*. 400 ppm)?

## Materials and Methods

### Plant growth conditions

*Laurus nobilis* L., *Drimys winteri* J.R. Forst. & G. Forst., *Osmunda regalis* L., *Agathis australis* (D. Don) Loudon, *Nageia nagi* Thunb. O. Kuntze, *Lepidozamia hopei* Regel, *L. peroffskyana* Regel*, Ginkgo biloba* L*. and Passiflora caerulae* L. were grown in 5 l square pots in a production glasshouse (Cambridge HOK) at UCD Rosemount Environmental Research Station, Dublin, Ireland under ambient Dublin light. Growth light intensity at canopy level (minimum 92 μmol m^−2^ s^−1^; maximum 386 μmol m^−2^ s^−1^; mean 221 μmol m^−2^ s^−1^), glasshouse ambient air temperature (minimum 22°C; maximum 31°C; mean 25.8°C) and relative humidity (minimum 42%; maximum 81%; mean 61.4%) were recorded hourly during the measurement period (27 June 2011–15 July 2011). *Protea eximia* (Knight) Fourc., *Punica granatum* L., *Greyia sutherlandii* Hook & Harv., *Colocasia esculenta* (L.) Schott, *Pelargonium* ‘Robert Fish’, *Citrus *× *sinensis* (L.) Osbeck, *Ceratonia silique* L., *Olea europea* L., *Manihot esculenta* Crantz and *Ricinus communis* L. were growing in the Mediterranean Collections of the Curvilinear Range glasshouse of the National Botanic Gardens Glasnevin, Ireland under ambient Dublin light. Light intensity at canopy level (304 ± 179 μmol m^−2^ s^−1^), glasshouse air temperature (minimum 19°C; maximum 31°C; mean 22°C) and relative humidity (minimum 47%; maximum 87%; mean 67%) were recorded hourly during the measurement period (17 June 2012–21 June 2012). All species were optimally watered and fed.

### Leaf gas exchange measurements

The *g*_op_ measurements were taken over a 13 d period in 2011 using a PP Systems CIRAS 2 portable infra‐red gas analyser (IRGA) equipped with a PP systems PLC 6 (U) leaf cuvette fitted with a rice plate (1.75 cm^2^) and a 5 d period in July 2012 using a SC‐1 leaf porometer (Decagon Devices, Pullman, WA, USA). Species growing at UCD Rosemount were measured with the IRGA set to the following: CO_2_ (400 μmol mol^−1^); light intensities were set to ambient; humidity was set to 60%; air flow to 150 μmol m^−2^ s^−1^ and vapour pressure deficit (VPD) *c*. 1 kPa. Species growing in the Mediterranean collections were measured using the SC‐1 leaf porometer. Each species is represented by measurements from two individuals per species, one to three leaves per individual, two to six measurements per leaf over the course of the day. This protocol was repeated for each leaf over a 5–13 d period resulting in a mean *g*_op_ based on between 42 and 72 individual measurements per species. This protocol, which we refer to here as the ‘variance protocol’ was carried out in order to capture the fullest range of variance in *g*_op_ for each species, growing in optimal soil water and nutrient conditions and under the same prevailing climate and light regime. In this respect the chosen methodology was nonconventional because typical protocols used to measure *g*_op_ attempt to minimize variance by standardizing the light and VPD conditions at the time of measurement and allowing a substantial time for *g*_op_ to stabilize to the new standardized measurement conditions. To ensure that our ‘variance protocol’ measured in 2011 and 2012 produced a robust mean *g*_op_ value for each species we repeated the entire experiment in October 2014 with a PP Systems CIRAS 2 portable IRGA following a standardized protocol as follows: the maximum operational stomatal conductance *g*_op(max)_ for each species was measured at saturating light intensity (determined from preliminary light curves for each species), a leaf temperature of 25°C and VPD *c*. 1 kPa. The intact leaves were left to equilibrate under optimal conditions for a minimum of 20 min until the increasing stomatal conductance reached a plateau. The *g*_op(max)_ of each leaf was then calculated as the average of three recordings taken upon full induction of stomatal opening.

### Morphological trait measurements and calculation of *g*_max_

Following completion of all the physiological measurements the leaves on which the *g*_op_ measurements were taken were removed from the plants in order to calculate the theoretical *g*_max_ using the following diffusion equation (Parlange & Waggoner, 1970; Franks & Beerling, 2009b);

$$g_{\max}=\frac{\frac{\text{dw}}{v}\cdot \text{SD}\cdot pa_{\max}}{pd+\frac{\pi }{2}\sqrt{pa_{\max}/\pi }}$$

where dw = diffusivity of water vapour at 25°C (0.0000249 m^2^ s^−1^) and *v* = molar volume of air (0.0224 m^3^ mol^−1^) are both constants, SD is stomatal density (m^−2^), *pa*_max_ is maximum stomatal pore area (m^2^) calculated as an ellipse using stomatal pore length (m) as the long axis and l/2 as the short axis; *pd* is stomatal pore depth (m) considered to be equivalent to the width of an inflated, fully turgid guard cell (Franks & Beerling, 2009a, 2009b).

A 1 cm^2^ leaf disc was taken for each leaf from the exact position of the *g*_op_ measurements and positive leaf impressions were taken from the abaxial leaf surface using dental impression material (Coltene PRESIDENT light body) followed by a negative impression using clear nail varnish. Five photomicrographs per leaf impression were recorded at ×200 magnification using a Leica (DMLB, Wetzlar, Germany) epifluorescent microscope. The SD was estimated for each photomicrograph using AcQuis (v.4.0.1.10; Syncroscopy Ltd, Cambridge, UK) by placing a 0.09 mm^2^ grid on the image and counting the number of stomata within the box and those touching two of the border lines and the corner where they intersect. The cuticle preparations were made for any species that did not yield clearly visible stomata from dental impressions using a 1 : 1 mixture of glacial acetic acid and hydrogen peroxide and heated at 65°C. Cuticles were rinsed in deionized water following 6–7 d of maceration, stained with safranin and mounted onto a slide containing glycerol gelatin. The stomatal pore length and guard cell width measurements were taken for 5–20 open stomata per photomicrograph in AcQuis. The vein density (*D*_v_) was determined for the same leaves on which conductance and stomatal density measurements were taken. The leaf tissue was bleached using 2.5 or 5% sodium hydroxide for 1–3 d, followed by sodium hypochlorite solution for 1 h to 1 d. The cleared leaves were then rinsed in deionized water and stained with mildly acidified Toluidine Blue (pH 5.5) for 2–10 min. Photomicrographs of the cleared leaves were taken at ×100 using a Nikon camera (Amsterdam, the Netherlands) stereomicroscope and six measurements of vein length in mm mm^−2^ area were made for each species in Auto‐Montage (v.5.03).

### Estimating saturated assimilation rate (*tA*_sat_) from *g*_max_

Instantaneous measurements of the assimilation rate (*A*; μmol m^−2^ s^−1^) at light intensities (PAR) of 700 μmol m^−2^ s^−1^ and ambient CO_2_ (400 μmol mol^−1^) were recorded alongside *g*_op_ measurements for a subset of eight species (*L. nobilis*,* D. winteri*,* O. regalis*,* A. australis*,* N. nagi*,* L. hopei*,* L. peroffskyana*,* G. biloba*) growing at UCD Rosemount Environmental research station using a CIRAS 2 portable IRGA (see *g*_op_ methodology earlier) over an 11 d period in 2011 (27 June 2011–15 July 2011). A logarithmic trend line was fitted to *A* and *g*_op_ for each of the eight species and used to estimate the theoretical saturated assimilation rates (*tA*_sat_) from *g*_max_ (see later Table 2). The reliability of the estimated *tA*_sat_ values was assessed by comparison with a new dataset of mean (of 10–12 replicates per species) *A*_sat_ and *A*_max_ measurements of the same eight species taken in 2013. The *A*_sat_ measurements were all performed at 400 ppm CO_2_ and a saturating light of 1000 μmol m^−2^ s^−1^ and all *A*_max_ measurements were performed at 2000 ppm CO_2_ and saturating light of 1000 μmol m^−2^ s^−1^ using a PP Systems CIRAS 2 portable infrared gas analyser. The flow rates were set at 150 μmol and all leaves were left in the chamber for *c*. 3 min (or until full photosynthetic induction). All measurements were carried out between 09:30 and 12:30 h.

## Results and discussion

### Scaling relationship between *g*_max_ and *g*_op_

**Figure 1:**
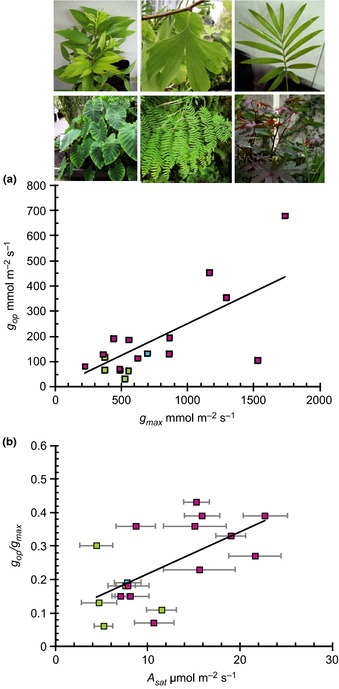
Relationship between stomatal and photosynthetic traits. (a) Scaling relationship between maximum theoretical stomatal conductance (*g*_max_) and mean operational stomatal conductance (*g*_op_) of five gymnosperms (green squares), one fern (blue squares) and 12 angiosperm (pink squares) species measured using the ‘variance protocol’ in glasshouse conditions over a 5–13 d period in 2011–2012. All data points represent mean *g*_op_ values of between 42 and 72 individual measurements per species regressed against *g*_max_ (*g*_op_ = 0.2507 *g*_max_; *r* ^2^ = 0.5446, *P *=* *0.00039). (b) Species *g*_op_ : *g*_max_ scaling relationships (± standard deviation) regressed against mean saturated photosynthetic rate (*A*_sat_) (*g*_op_ : *g*_max_ = 0.0125*A*_sat_ + 0.093; *r* ^2^ = 0.36652; *P *=* *0.007758). Examples of species studied from left to right include *Nageia nagi*,* Ginkgo biloba* and *Lepidozamia peroffskyana* (upper panel) and *Colocasia esculenta*, *Osmunda regalis* and *Ricinus communis* (lower panel).

The best fit scaling relationship between *g*_max_ and *g*_op_ for 12 angiosperm, five gymnosperm and one fern species investigated in this study was found to be *g*_op_ = 0.25 *g*_max_ (*r* ^2^ = 0.5446, *P *=* *0.00039) (Fig. 1a) which is in good agreement with the previously reported results from single species studies such as *Eucalyptus* (*g*_op_ *c*. 0.2 *g*_max_; Franks *et al*., 2009) and *Arabidopsis* (*g*_op_ = 0.2 *g*_max_; Dow *et al*., 2014) but lower than some purely experimental based reports (*g*_op_ = 0.5 *g*_max_−0.03; Franks *et al*., 2014). The results of this study are the most comprehensive, to date, in terms of the number of species investigated and confirm that, on average, plants operate at a stomatal conductance that is only a fraction (*c*. 25%) of their theoretical maximum anatomical limits when growing in field and glasshouse conditions (Fig.  1a, Supporting Information Fig. S1).

Despite the apparent convergence in the ratio of *g*_op_ : *g*_max_ from three independent studies (Franks *et al*., 2009; Dow *et al*., 2014; this work) considerable variability exists at the individual species level which cannot be easily explained by biogeography, habit, ecology or partitioned by phylogenetic group (e.g. angiosperm vs gymnosperm; Table S1, Fig. S2). For example, stomatal conductivities of the tropical crops *R. communis* (castor oil), *Ceratonia siliqua* (carob) and *M. esculenta* (cassava) occasionally reach 80% of their respective *g*_max_, but another important crop *C. esculenta* (taro) operates further under its theoretical *g*_max_ than *G. biloba*, a ‘living fossil’ gymnosperm (Fig. S2). At the opposite end of the scale, *N. nagi*, a broad‐leafed temperate conifer from Japan and two woody Magnoliids from contrasting climates, *L. nobilis* (Mediterranean) and *D. winteri* (temperate rain forest) all conduct at < 20% of their theoretical potential (*g*_max_ ≤ 0.2 *g*_op_, Fig. S2). The species‐level scaling relationship between *g*_max_ and *g*_op_ could also not be predicted based on stomatal density (*R* ^2^ = 0.0166), stomatal pore length (*R* ^2^ = 0.0862) or pore depth (*R* ^2^ = 0.2927) (Table 1). However, there was a tendency among all species belonging to more recently derived lineages to utilize a much greater proportion of the theoretical *g*_max_ than species with deep phylogenetic origins (Table S2). This pattern, although not statistically significant (*g*_op_ : *g*_max_ vs generic stem age: *R* ^2^ = 0.1662, Table S2) suggested that perhaps older lineages were constrained by some other aspect of their morphology/anatomy, such as vein density than more recently derived species. It is also possible that aspects of stomatal complex morphology, anatomy and/or chemistry (e.g. guard cell lignification, presence/absence of stomatal ledges and subsidiary cells) not considered here may also play a role in the range of interspecies variability observed between *g*_op_ : *g*_max_ (Franks & Farquhar, 2007).

**Table 1:** Mean (± standard deviation) stomatal density (SD), geometry, conductance and vein density data

Species	SD mm^−2^	Pore length μm	Pore depth μm	Maximum *g*_op_ : *g*_max_	*g*_max_ mmol m^−2^ s^−1^	Average *g*_op_ mmol m^−2^ s^−1^	Maximum *g*_op_ mmol m^−2^ s^−1^	Average *g*_op_ : *g*_max_	*D*_v_ mm mm^−2^
*Lepidozamia hopei*	39 ± 3.9	26.2 ± 1.8	14.12 ± 5.9	0.398	374	66 ± 29.8	149	0.18	0.93
*Nageia nagi*	104 ± 9.7	15.9 ± 0.8	11.20 ± 1.44	0.146	526	31 ± 20.9	77	0.06	1.02
*Agathis australis*	95 ± 15.4	19.4 ± 0.8	15.09 ± 1.52	0.308	554	64 ± 31.5	171	0.11	1.13
*L. peroffskyana*	40 ± 1.3	27.7 ± 1.4	9.65 ± 1.63	0.334	491	65 ± 41.5	164	0.13	1.33
*Ginkgo biloba*	51 ± 15.6	19.9 ± 1.8	10.57 ± 2.83	0.692	374	117 ± 68.3	259	0.31	1.37
*Osmunda regalis*	55 ± 8.0	32.7 ± 4.0	17.02 ± 3.2	0.375	698	132 ± 66.0	262	0.19	3.27
*Drimys winteri*	131 ± 11.9	18.3 ± 2.9	10.19 ± 1.05	0.293	863	130 ± 44.1	253	0.15	4.87
*Protea eximia*	76 ± 6.0	14.1 ± 2.3	8.9 ± 1.0	0.590	361	129 ± 115.2	213	0.36	2.6
*Punica granatum*	80 ± 1.0	7.5 ± 1.1	3.8 ± 0.5	0.765	226	81 ± 43.0	173	0.36	3.0
*Greyia sutherlandii*	83 ± 11	17.7 ± 3.3	8.8 ± 0.9	0.714	559	186 ± 95.9	399	0.33	3.2
*Laurus nobilis*	205 ± 29.8	19.2 ± 1.9	9.14 ± 1.19	0.164	1529	105 ± 88.7	250	0.07	5.71
*Colocasia esculenta*	81 ± 10	13.3 ± 2.4	4.4 ± 1.3	0.530	487	71 ± 58.1	258	0.15	4.4
*Pelargonium* rf	63 ± 7	15.7 ± 2.7	5.3 ± 0.7	0.966	443	191 ± 89.1	428	0.43	4.6
*Citrus sinensis*	390 ± 12	6.3 ± 1.0	6.4 ± 1.0	0.364	626	113 ± 45.3	228	0.18	5.1
*Ceratonia siliqua*	135 ± 15	12.9 ± 1.8	3.8 ± 0.6	0.834	1292	354 ± 194.2	1077	0.27	6.2
*Olea europea*	345 ± 11	6.2 ± 0.7	2.7 ± 0.4	0.481	865	195 ± 110.1	416	0.23	6.2
*Manihot esculenta*	180 ± 11	16.7 ± 1.8	8.0 ± 1.3	0.811	1164	452 ± 180.4	944	0.39	6.7
*Ricinus communis*	163 ± 19	15.1 ± 1.8	5.3 ± 0.8	0.984	1736	677 ± 350.0	1708	0.39	8.7

*g*_op_, operational stomatal conductance; *g*_max_, maximum theoretical stomatal conductance; *D*_v_, leaf vein density.

To investigate the potential underlying reasons for interspecies variability, *g*_op_ : *g*_max_ ratios were plotted against saturating assimilation (*A*_sat_) rates for all 18 species investigated. A significant positive relationship was observed (*r* ^2^ = 0.36652, *P *=* *0.0077) suggesting that species with innately higher net photosynthetic rates use more of their maximum anatomical potential in terms of gas exchange than species with lower *A*_sat_ values (Fig. 1b). This is an important finding in the context of palaeoecophysiological studies as it provides further constraints on our capacity to estimate gas exchange rates from fossil stomatal anatomical data. It also has a bearing on palaeo‐CO_2_ estimates that are based in large part on accurate estimates of the fossil plant *g*_op_ such as that of Franks *et al*. (2014). Based on the analysis presented in Fig. 1(b), a *g*_op_ : *g*_max_ ratio of > 0.25 should be assigned to fossil taxa with high photosynthetic rates (which can be inferred from high vein densities (*D*_v_) (Boyce & Zwieniecki, 2012) or short vein to stomatal distances (Brodribb *et al*., 2007). By contrast *g*_op_ : *g*_max_ ratios of < 0.25 should be assigned to fossil taxa if other anatomical data indicate that they had low photosynthetic rates.

### Coordination of stomatal and vein density evolution

**Figure 2:**
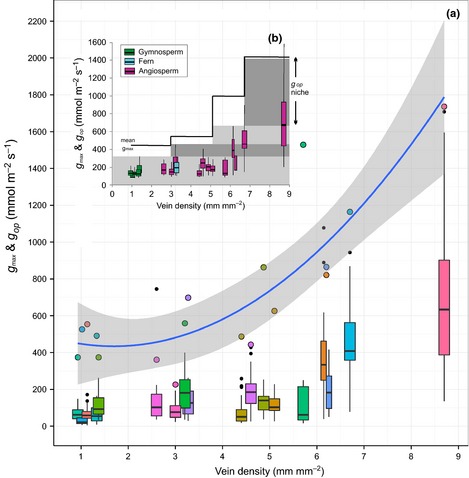
Examination of relationships between stomatal and leaf vein traits. (a) Relationship between vein density (*D*_v_, mm mm^−2^) and both operational stomatal conductance (*g*_op_; coloured boxplots based on variance protocol from 2011 to 2012) and theoretical stomatal conductance (*g*_max_; coloured dots) of 12 angiosperms (ang.), five gymnosperms (gym.) and one fern (fn.). From left to right species are: *Lepidozamia hopei* (gym., cycad), *Nageia nagi* (gym., conifer), *Agathis australis* (gym., conifer), *L. peroffskyana* (gym., cycad), *Ginkgo biloba* (gym.), *Protea examina* (ang.), *Punica granatum* (ang.), *Greyia sutherlandii* (ang.), *Osmunda regalis* (fn.), *Colocasia esculenta* (ang.), *Pelargonium* ‘Robert Fish’ (ang.), *Drimys winteri* (ang.), *Citrus sinensis* (ang.), *Laurus nobilis* (ang.), *Ceratonia siliqua* (ang.), *Olea europea* (ang.), *Manihot esculenta* (ang.), *Ricinus communis* (ang.). Regression line (blue) is a second order polynomial between *g*_max_ and *D*_v_ (*r* ^2^ = 0.741; *y *=* *27.574*x* ^2^ − 93.365*x *+ 512.84). Small black dots are statistical outlier *g*_op_ values. *g*_op_ boxplots illustrate the 25% and 75% quartiles (top and bottom of box), median values (horizontal bar) and whiskers represent 2nd and 98th percentiles for each species based on between 42 and 72 individual measurements. (b) Inset figure illustrates the theoretical expansion of ecophysiological (*g*_op_) space (grey horizontal bars) with increasing *g*_max_ and *D*_v_. Solid line is mean *g*_max_ for *D*_v_ 0–3, > 3–5, > 5–7 and > 7–9 mm mm^−2^.

This study is the first detailed examination of the relationships between *g*_op_, *g*_max_ and *D*_v_. A strong positive correlation was observed between vein density (*D*_v_) and *g*_op_ (*R* ^2^ = 0.8314; *y *=* *14.379*x* ^2^ − 65.238*x *+ 139.79) (Fig. 2, Table 1) which indicates close coordination of stomatal and vein density evolution in agreement with previous studies (Sack *et al*., 2003; Brodribb *et al*., 2007; Boyce *et al*., 2009; Feild *et al*., 2011b). We also demonstrate a significant positive correlation between vein density and theoretical *g*_max_ (*R* ^2^ = 0.741; Fig. 2) suggesting that the evolution of high vein density angiosperms during the Cretaceous period may have facilitated a rise in their theoretical maximum stomatal conductivities. This would have allowed an increase in the average operational conductivities of angiosperms with *D*_v_s over 4 mm mm^−2^ (Fig. 2). More importantly, the results also show that the margin of difference between *g*_max_ and *g*_op_ increases with *D*_v_ (Fig. S3). This suggests that the evolution of high vein densities also dramatically increased the range of *g*_op_ in angiosperms providing them with significantly greater plasticity and flexibility to maximize CO_2_ uptake and water loss when conditions were optimal (Fig. 2). Our analysis shows that species with vein densities of ≤ 4.0 mm mm^−2^ have mean *g*_op_ values *c*. 400 mmol m^−2^ s^−1^ below their respective theoretical *g*_max_ values, whereas the majority of species with *D*_v_ of 6 mm mm^−2^ and greater are typically operating between 600 and 1000 mmol m^−2^ s^−1^ below their theoretical maximum potential (*g*_max_) (Fig. 2). Our study therefore supports the ‘vein density hypothesis’ (Boyce *et al*., 2009; Brodribb & Feild, 2010; Feild *et al*., 2011a) that proposes a transformative influence of high vein density on conductive capacity of angiosperms compared with gymnosperms but goes further to show that the coordination of high vein density and high *g*_max_ also dramatically increased the dynamic operational conductance range of angiosperms (as indicated by wide interquartile *g*_op_ ranges in Fig. 2b) compared with other seed plant groups. Indeed the examination here of scaling relationships between *g*_max_, *g*_op_ and *D*_v_ (Fig. 2) helps to explain the apparent coordinated surge in both *D*_v_ (Boyce *et al*., 2009; Feild *et al*., 2011a) and modelled *g*_op_ (Fig. 7 in Franks & Beerling, 2009a,b) *c*. 100 Mya in the mid Cretaceous, the latter of which Franks & Beerling attribute to falling atmospheric CO_2_.

The advantage of a significantly expanded *g*_op_ range in angiosperms is that it likely conferred greater ecophysiological plasticity allowing species with a high vein density and high *g*_max_ to operate within a much wider ‘ecophysiological niche space’. This in turn may have provided an opportunity for a population to segregate resource use by stomatal conductance. The plasticity of behaviour would only be possible in high *D*_v_ and high *g*_max_ species because species with low *D*_v_ and low *g*_max_, as illustrated in Fig. 2(b), have very constrained and overlapping *g*_op_ ranges. Following this reasoning it could be argued that on evolutionary timescales increasing vein densities in angiosperms not only expanded the capacity for increased uptake of CO_2_ in exchange for water (Boyce *et al*., 2009; Brodribb & Feild, 2010; Feild *et al*., 2011a), but also expanded the breadth of ecophysiological niche space on which selection could act. Stomatal control of *g*_op_ in a patchy landscape may therefore have enabled different individuals within the same species, or different species within the same community, to share rather than compete for available resources (N, P, H_2_O) by controlling how close or far away the species operated from maximum theoretical (*g*_max_) limits. Increased landscape complexity and ‘patchiness’ in terms of available niches has been suggested for the Cretaceous period (Coiffard *et al*., 2012). This potential mechanism for angiosperm speciation is demonstrated conceptually in Fig. 2(b) where the number of theoretical ‘stomatal conductance niches’ are shown to increase with increasing *g*_max_ and *D*_v_. By contrast, all species with a low *D*_v_ have narrow and overlapping *g*_op_ ranges. Support for the concept of increasing ecological complementarity via an expansion of stomatal conductance niches comes from stable carbon and oxygen isotopic studies (Moreno‐Gutiérrez *et al*., 2012). These show evidence of consistent segregation of ‘ecophysiological niche space’ among coexisting species to maximize community level plant water use efficiency (Moreno‐Gutiérrez *et al*., 2012). The concept of wide plasticity in angiosperms compared with gymnosperms has been demonstrated for many traits such as genome size (Leitch & Leitch, 2013), post‐disturbance regeneration time (Midgley & Bond, 1991), pollination to fertilization interval (Cernusak *et al*., 2009). We suggest that plasticity of operational stomatal conductance is an additional example of behavioural flexibility that angiosperms may have capitalized on. We acknowledge, however, that when considering the role of stomata in ecological interactions among species the speed of stomatal opening/closing response and regulation of stomata by environmental factors and at the signalling level (Hetherington & Woodward, 2003; Franks & Farquhar, 2007; Brodribb *et al*., 2009; Brodribb & McAdam, 2011; Lawson *et al*., 2011; Lawson & Blatt, 2014; McAdam & Brodribb, 2015) are likely equally important as wide plasticity in *g*_op_ and high absolute conductive capacity in determining a species competitive ability.

Within species there is a tendency for increased *g*_op_ to be achieved by decreasing stomatal size and increasing density (Hetherington & Woodward, 2003; Franks *et al*., 2009). The present study suggests that this tendency may not apply across species as no significant correlations were observed between *g*_op_ and stomatal pore area (*r* ^2^ = 0.0042) or depth (*r* ^2^ = 0.0525) and a complex relationship was observed with stomatal density (SD), where both operational and theoretical conductivities increased with increasing SD (*r* ^2^ = 0.6675, *g*_max_ and SD; *r* ^2^ = 0.3255, *g*_op_ and SD) up to a threshold of *c*. 250 stomata mm^−2^, after which they declined sharply despite increasing SD (Fig. S4). This suggests that the observed pattern of steeply increasing *g*_max_ and *g*_op_ in species with high vein densities (Fig. 2a) was achieved by multiple combinations of different stomatal density and pore geometries and was not exclusively driven by high densities of small stomata as previously predicted (Franks *et al*., 2009; de Boer *et al*., 2012). The lack of a significant relationship between the stomatal pore area and *g*_op_ may be due to the small sample size of just 18 species. Equally, however, this may reflect different weighting of the functional roles of stomata in the species analysed as part of this study. Stomata have three primary functional roles (Raven, 2002): they optimize CO_2_ uptake against water loss; they are involved in thermoregulation of leaves via conductive cooling and they provide protection against catastrophic embolisms. Optimization of gas exchange in environments that suffer water deficit may select for species with high densities of small stomata (Franks *et al*., 2009; de Boer *et al*., 2012), however, the requirement for leaf cooling in hot climates with high light intensities may select for species with moderate densities of mid‐sized stomata (e.g. *R. communis*, Table 1).

**Figure 3:**
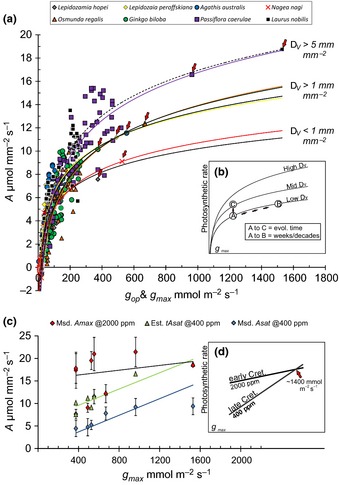
Examination of relationships between stomatal conductance and assimilation. (a) Assimilation rate (*A*) vs operational stomatal conductance (*g*_op_, coloured symbols) and maximum theoretical stomatal conductance (*g*_max_, red arrow) collected in 2011 and 2012 for a range of species with low (≤ 1.00 mm mm^−2^), medium (> 1–3.5 mm mm^−2^) and high (> 5.0 mm mm^−2^) vein densities (*D*_v_). See Table 2 for *r* ^2^ values and trendline equations for each species. (b) Illustrates potential pathways to increasing assimilation rate. A–C would take place on evolutionary timescales, whereas A–B can occur within the lifetime of an individual plant by increasing stomatal density and/or pore geometry. (c) A comparison of the relationships between measured *A*_sat_ (± standard deviation) and *g*_max_ (*A*_sat_ = 0.0092, *g*_max_, *r* ^2^ = 0.896), estimated saturated assimilation rate (*tA*_sat_) and *g*_max_ (*tA*_sat_ = 0.009 *G*_max_ + 5.972, *r* ^2^ = 0.8766) and measured *A*_max_ and *g*_max_ (*A*_max_ = 0.0026 *G*_max_ + 15.109, *r* ^2^ = 0.0566). Inset figure (d) is a stylized representation of data in (c) illustrating that low *g*_max_ would likely not have imposed a diffusional limitation on assimilation rate in the high CO_2_ world of early Cretaceous (*c*. 2000 ppm) but would likely induce a severe diffusional limitation by the late Cretaceous when CO_2_ levels had dropped to levels close to modern ambient (*c*. 400 ppm).

The trend of increasing disparity between *g*_max_ and *g*_op_ in species with a high vein density was unexpected. We have interpreted the trend in terms of a means to expand the dynamic range of ecophysiological behaviour of high vein density species. An alternative interpretation is that the ecophysiological behaviour of modern extant species is somehow a legacy of the palaeoatmospheric conditions under which they radiated. Gymnosperms as a group were ecologically dominant during the early and middle Mesozoic when atmospheric CO_2_ concentrations were on average five times higher than today (e.g. *c*. 2000 μmol mol^−1^). Angiosperms on the other hand underwent their greatest radiation as CO_2_ concentrations declined through the Cretaceous period. It is widely theorized (McElwain *et al*., 2005; Brodribb & Feild, 2010; Feild *et al*., 2011a) but not universally accepted (Boyce & Zwieniecki, 2012) that the declining Cretaceous atmospheric CO_2_ concentration from *c*. 2000 to *c*. 400 μmol mol^−1^ contributed to the rise in angiosperms over gymnosperms because angiosperms uniquely developed traits that would enable them to maintain carbon gain under ‘CO_2_ starvation’. Next therefore, we quantified the direct photosynthetic advantage of increasing *g*_max_ via an increase in stomatal density and/or stomatal pore geometry under both ambient (400 ppm) and simulated Cretaceous (2000 ppm) CO_2_ (Fig. 3a,c).

### Maximum photosynthetic capacity is regulated by coordination of *g*_max_ and *D*_v_

Theoretical saturated assimilation rates (which we refer to here as *tA*_sat_) were estimated for a subset of eight species (one fern, five gymnosperms and two angiosperms) based on their respective *g*_max_ values fitted to *A* : *g*_op_ curves collected using gas analysis in 2011 (see the Materials and Methods section). Our results suggest that although stomatal limitation on photosynthesis may be small within species (Farquhar & Sharkey, 1982; Hetherington & Woodward, 2003), comparison across species demonstrates that differences in *g*_max_ can impose a significant constraint on the assimilation rate (Table 2). Species with low *g*_max_ (*c*. 400 mmol m^−2^ s^−1^), for example *L. hopei* (*D*_v_ = 0.93 mm mm^−2^, *tA*_sat_ = 7.56 μmol m^−2^ s^−1^) and *G. biloba* (*D*_v_ = 3.05 mm mm^−2^, *tA*_sat_ = 10.15 μmol m^−2^ s^−1^), have *tA*_sat_ values capped at *c*. 11 μmol m^−2^ s^−1^ under current ambient CO_2_ concentrations (Fig. 3a, Table 2). Species with moderate *g*_max_ values (*c*. 700 mmol m^−2^ s^−1^) such as *A. australis* (*D*_v_ = 1.13 mm mm^−2^, *tA*_sat_ = 10.15 μmol m^−2^ s^−1^) and *O. regalis* (*D*_v_ = 3.05 mm mm^−2^, *tA*_sat_ = 10.15 μmol m^−2^ s^−1^) have slightly higher assimilation rates of *c*. 12 μmol m^−2^ s^−1^, whereas species with the highest *g*_max_ values of over 1000 mmol m^−2^ s^−1^ have the correspondingly highest assimilation rates (e.g. *L. nobilis*;* D*_v_ = 5.71 mm mm^−2^, *tA*_sat_ = 18.74 μmol m^−2^ s^−1^ and *P. caerulae D*_v_ = 12.36 mm mm^−2^, *tA*_sat_ = 16.58 μmol m^−2^ s^−1^) (Fig. 3a, Table 2).

**Table 2:** Estimated and measured assimilation rates

Species	*g*_max_ mmol m^2^ s^−1^	Measured *A*_sat_ at 400 ppm μmol^−1^ mm^2^ s^−1^	Measured *A*_max_ at 2000 ppm μmol^−1^ mm^2^ s^−1^	Est. *tA*_max_ at 400 ppm V μmol^−1^ mm^2^ s^−1^	Model to estimate *tA*_max_ from *g*_max_
*Lepidozamia hopei (G)*	374	7.63 ± 1.06	17.20 ± 4.00	7.56	*tA*_max_ = 1.9624log_e_ (*g*_max_)−4.06 (*r* ^2^ 0.4475; *P *<* *0.001)
*Lepidozamia peroffskiana (G)*	491	4.73 ± 1.91	8.87 ± 0.98	11.17	*tA*_max_ = 3.1225log_e_ (*g*_max_)−8.18 (*r* ^2^ 0.8111; *P *<* *0.001)
*Nageia nagi (G)*	526	5.24 ± 1.02	19.34 ± 2.00	9.11	*tA*_max_ = 2.4814log_e_ (*g*_max_)−6.44 (*r* ^2^ 0.793; *P *<* *0.001)
*Agathis australis (G)*	554	11.5 ± 1.59	20.80 ± 3.70	11.55	*tA*_max_ = 3.1225log_e_ (*g*_max_)−8.18 (*r* ^2^ 0.6587; *P *<* *0.001)
*Osmunda regalis (F)*	669	7.79 ± 1.44	12.00 ± 2.00	12.36	*tA*_max_ = 3.9086log_e_ (*g*_max_)−13.07 (*r* ^2^ 7003; *P *<* *0.001)
*Gingko biloba (G)*	374	4.44 ± 1.80	17.60 ± 3.30	10.15	*tA*_max_ = 3.7973log_e_ (*g*_max_)−12.35 (*r* ^2^ 0.7109; *P *<* *0.001)
*Laurus nobilis (A)*	1529	9.37 ± 1.86	18.29 ± 0.26	18.74	*tA*_max_ = 4.2584log_e_ (*g*_max_)−12.48 (*r* ^2^ 0.9055; *P *<* *0.001)
*Passiflora caerulae (A)*	963	9.20 ± 1.90	21.25 ± 3.29	16.58	*tA*_max_ = 4.4852log_e_ (*g*_max_)−14.24 (*r* ^2^ 0.7092; *P *<* *0.001)

*g*_max_, maximum theoretical stomatal conductance; *A*_sat_, saturated assimilation rate; *A*_max_, maximum assimilation rate. *n *=* *10–12; errors, ± SD; G, gymnosperm; A, angiosperm; F, fern.

The interspecies comparison in Fig. 3, highlighted by the inset figure (Fig.  3b), illustrates that assimilation rates can be enhanced by one of two possible routes: (1) by increasing *g*_max_ and holding vein density (*D*_v_) constant (Fig. 3b); or (2) by maintaining the same *g*_max_ and increasing *D*_v_. The stomatal density and geometry are highly responsive to atmospheric CO_2_ concentration on time‐scales of weeks to decades (Wagner *et al*., 1996; Haworth *et al*., 2013) but vein densities are much less responsive to CO_2_, despite being highly responsive to many other environmental factors (Uhl & Mosbrugger, 1999). This implies that increases in assimilation rate via developmental and/or morphological processes (underlying biochemistry is not considered here but may play a role) could be rapidly achieved within an individual plant or population by increasing *g*_max_, but could only be achieved on evolutionary timescales by increasing vein density.

It also highlights how ‘transformative’ (Boyce *et al.,* 2009) increasing the vein density was for angiosperms compared with gymnosperms because although species with a low *D*_v_ can incrementally enhance assimilation rates with relatively small changes in *D*_v_ *or g*_max_, a doubling of assimilation rates via morphological/developmental change can only be achieved by increasing *D*_v_ above 5 mm mm^−2^ (Fig. 3 inset). It is argued that both *D*_v_ and *g*_max_ are coordinated at the leaf level by evolutionary controls on cell size (Brodribb *et al*., 2013) which imply that simple modification to cell size, perhaps through whole genome duplication (which is common among angiosperms but not gymnosperms; Van de Peer *et al*., 2009) would offer a means of doubling *D*_v_ and *g*_max_ in a coordinated way. The comparison here of *Passiflora* and *Laurus* (Fig. 3a) also illustrates that once a *D*_v_ threshold of 5 has been passed, subtle changes in *g*_max_ can have an equally important control on the assimilation rate, as do changes in *D*_v_ under current ambient CO_2_. This observation was predicted by the model of de Boer *et al*. (2012).

Our data show an unequivocal advantage of angiosperm species with high *D*_v_ and/or high *g*_max_ at modern ambient CO_2_ concentrations of *c*. 400 μmol mol^−1^ (Fig. 3a). In order to test further the hypothesis that high *g*_max_ in species with a high vein density conferred an advantage to angiosperms under declining Cretaceous atmospheric CO_2_ concentration, we also examined whether the photosynthetic advantage of high *D*_v_ and *g*_max_ could be lost under elevated CO_2_ conditions of 2000 μmol mol^−1^, similar to those of the early Cretaceous when gymnosperms were the dominant ecological element in the majority of world biomes. Maximum (*A*_max_) and saturating (*A*_sat_) assimilation rates were measured for the same subset of eight species (one fern, five gymnosperms and two angiosperms) under saturating light (1000 μmol m^−2^ s^−1^) at 2000 μmol mol^−1^ and 400 μmol mol^−1^ CO_2_, respectively (Tables 2, S2). The results show that an elevated atmospheric CO_2_ concentration only modestly raises *A*_max_ above *A*_sat_ in the studied angiosperms (by 113 ± 18%) but had a profound effect on gymnosperms, raising the *A*_max_ by 173 ± 46% above *A*_sat_ (Fig. 3c, Table 2). This is consistent with the modelled differences between evergreens and deciduous taxa where species with robust leaves and high mesophyll resistance showed a significant increase in *A* and water use efficiency under elevated CO_2_ (Niinemets *et al*., 2011). These results indicate that the photosynthetic advantage conferred by high *D*_v_ and high *g*_max_ under modern ambient atmospheric CO_2_ levels, was likely to be completely lost in the elevated [CO_2_] world of the early Cretaceous (Fig. 3c).

Elevated CO_2_ reduces the diffusional limitation of stomata on assimilation rates in any species with low *g*_max_ values. This is best demonstrated by the substantial rise (by 173%) in *A*_max_ values of the gymnosperms studied here when exposed to elevated [CO_2_]. Based on our analysis it is likely that early Cretaceous gymnosperm species with low *g*_max_ and low *D*_v_, similar to the cycad, ginkgo and conifers examined in this study, would have possessed assimilation rates as high as or higher than extant angiosperms (Fig. 3c). By contrast, when the same species were subjected to CO_2_ levels more similar to those of the late Cretaceous (*c*. 400 μmol mol^−1^; Barclay *et al*., 2010), a severe diffusional penalty was imposed. These results on modern extant taxa predict that palaeospecies with *g*_max_ values at or below a threshold level of *c*. 1400 mmol mm^−2^ s^−1^ (Fig. 3d) would not have been able to maintain assimilation rates equivalent to species possessing high *g*_max_ values under Cretaceous atmospheric CO_2_ decline. In a palaeolandscape where other environmental conditions were optimal for growth lower assimilation rates would therefore undermine the competitive ability of low *g*_max_ species. A detailed site‐specific analysis of fossil leaf *g*_max_ values in Cretaceous angiosperms vs gymnosperms is now required to examine when and if angiosperms crossed this critical *g*_max_ threshold of *c*. 1400 mmol mm^−2^ s^−1^ and whether it coincided with the first occurrence of leaves with vein densities > 5 mm mm^−2^ as would be predicted if both evolved in coordination. As atmospheric CO_2_ levels continue to rise over the next century the competitive landscape of gymnosperms and angiosperms may shift again to a level playing field where diffusional limitation on assimilation is reduced for species with low densities of stomata and veins.

### Conclusions

Despite extensive field surveys of *g*_op_ across different plant species and environmental gradients (Körner, 1994; Schulze *et al*., 1994; Lin *et al*., 2015) understanding of the scaling relationship between *g*_op_ and *g*_max_ is limited to just a handful of species (Beerling *et al*., 1998; Franks *et al*., 2009; Dow *et al*., 2014). Further understanding of this relationship would enable anatomical traits to be linked to function, providing a means of tracking palaeophysiological responses over geological time. We have demonstrated that, on average, species growing in glasshouse conditions conduct H_2_O and CO_2_ through stomatal pores at *c*. 25% of their theoretical maximum limits (*g*_op_ = 0.25 *g*_max_) determined by stomatal geometry and density (Fig. 1). Wide variability in mean *g*_op_ : *g*_max_ is, however, apparent and appears to be tightly correlated with *A*_sat_.

A strong positive correlation was observed between vein density (*D*_v_) and *g*_op_ (*r* ^2^ = 0.8314), and between vein density and theoretical *g*_max_ (*r* ^2^ = 0.741), both of which indicate close coordination of stomatal and vein density evolution. Our study elaborates on the ‘vein density hypothesis’ (Boyce *et al*., 2009; Brodribb & Feild, 2010; Feild *et al*., 2011a) by proposing that the coordinated evolution of high vein density and high *g*_max_ in angiosperms dramatically increased their range of dynamic operational conductance compared with gymnosperm ancestors. This likely conferred greater ecophysiological plasticity to angiosperms allowing species with a high *g*_max_ and *D*_v_ to operate within a much wider ecophysiological niche space, which in turn provided an opportunity for a population to segregate resource use by stomatal conductance. Our study also supports the ‘Cretaceous carbon starvation hypothesis’; we have demonstrated that the evolution of unique traits such as high *D*_v_ and *g*_max_ in angiosperms conferred them with a competitive advantage over gymnosperms by facilitating higher assimilation rates as atmospheric CO_2_ declined, but also by greatly expanding the ecophysiological niche space in which they could operate.

## Supporting information

Please note: Wiley Blackwell are not responsible for the content or functionality of any supporting information supplied by the authors. Any queries (other than missing material) should be directed to the *New Phytologist* Central Office.

**Fig. S1** A comparison of the linear relationships between average *g*_op_ and anatomical *g*_max_ when *g*_op_ is measured using the ‘variance protocol’ with a porometer vs the standardized protocol using an IRGA.
**Fig. S2** Range of scaling relationships observed between mean *g*_op_ and theoretical *g*_max_ and maximum *g*_op_ and *g*_max_ for gymnosperms, a fern and angiosperms.
**Fig. S3** Graph showing that the divergence between *g*_max_ and *g*_op_ increases with increasing *D*_v_.
**Fig. S4** Graph illustrating relationship between maximum theoretical stomatal conductance (*g*_max_) and stomatal density (SD) and between operational stomatal conductance (*g*_op_) and SD.
**Table S1** Estimated stem and crown ages of species lineages studied
**Table S2** Species investigated and number of replicates in repeat analysis dataset October 2015 *g*_op(max)_
