# Supplementary material for: Using modern plant trait relationships between observed and theoretical maximum stomatal conductance and vein density to examine patterns of plant macroevolution
Source: New Phytol. 2015 Jul 31;209(1):94–103. doi: 10.1111/nph.13579 (PMC5014202; doi:10.1111/nph.13579)
Supplement: Supplementary file 1 — Fig. S1 A comparison of the linear relationships between average g op and anatomical g max when g op is measured using the ‘variance protocol’ with a porometer vs the standardized protocol using an IRGA. Fig. S2 Range of scaling relationships observed between mean g op and theoretical g max and maximum g op and g max for gymnosperms, a fern and angiosperms. Fig. S3 Graph showing that the divergence between g max and g op increases with increasing D v. Fig. S4 Graph illustrating relationship between maximum theoretical stomatal conductance (g max) and stomatal density (SD) and between operational stomatal conductance (g op) and SD. Table S1 Estimated stem and crown ages of species lineages studied Table S2 Species investigated and number of replicates in repeat analysis dataset October 2015 g op(max) [file NPH-209-94-s001.pdf]

New Phytologist

***New Phytologist* Supporting Information Figs S1–S4 and Table S1**

Article title: Using modern plant trait relationships between observed and theoretical maximum stomatal conductance and vein density to examine patterns of plant macroevolution

Authors: Jennifer C. McElwain, Charilaos Yiotis and Tracy Lawson

Article acceptance date: 27 June 2015

The following Supporting Information is available for this article:

**Fig. S1** A comparison of the linear relationships between average $g_{\mathrm{op}}$ and anatomical $g_{\mathrm{max}}$ when $g_{op}$ is measured using the ‘variance protocol’ with a Porometer vs the standardized protocol using an IRGA.

**Fig. S2** Range of scaling relationships observed between mean $g_{op}$ and theoretical $g_{max}$ and maximum $g_{op}$ and $g_{max}$ for gymnosperms, a fern and angiosperms.

**Fig. S3** Graph showing that the divergence between $g_{max}$ and $g_{op}$ increases with increasing $D_v$.

**Fig. S4** Graph illustrating relationship between maximum theoretical stomatal conductance ($g_{max}$) and stomatal density (SD) and between operational stomatal conductance ($g_{op}$) and SD.

**Table S1** Estimated stem and crown ages of species lineages studied

**Table S2** Species investigated and number of replicates in repeat analysis dataset October 2015 $g_{op(\mathrm{max})}$

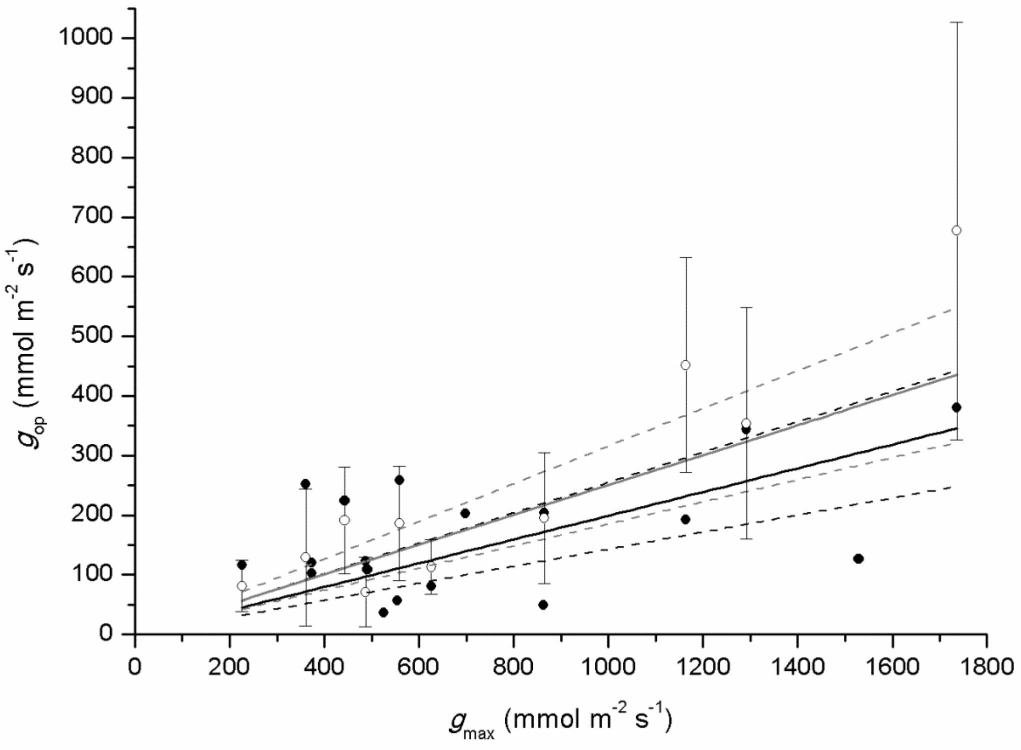


**Fig. S1** Linear relationships (solid lines) and 95% confidence levels (dashed lines) between average $g_{op}$ measured using the 'variance protocol' (grey colour, $P = 3.1\times10^{-7}$) / $g_{\mathrm{op(max)}}$ following the standardized protocol (black colour, $P = 8.6\times10^{-7}$) and anatomical $g_{\mathrm{max}}$ of all test species. Each point represents one species and is the mean ± standard deviation of 42–72 $g_{\mathrm{op}}$ measurements (variance protocol, open circles) or 6–14 $g_{\mathrm{op(max)}}$ measurements (standardized protocol, closed circles). Average $g_{op} = 0.2507 \times g_{max}$ (variance protocol: $r^2 = 0.5445$). $g_{op(max)} = 0.1989 \times g_{max}$ (standardized protocol : $r^2 = 0.0769$).

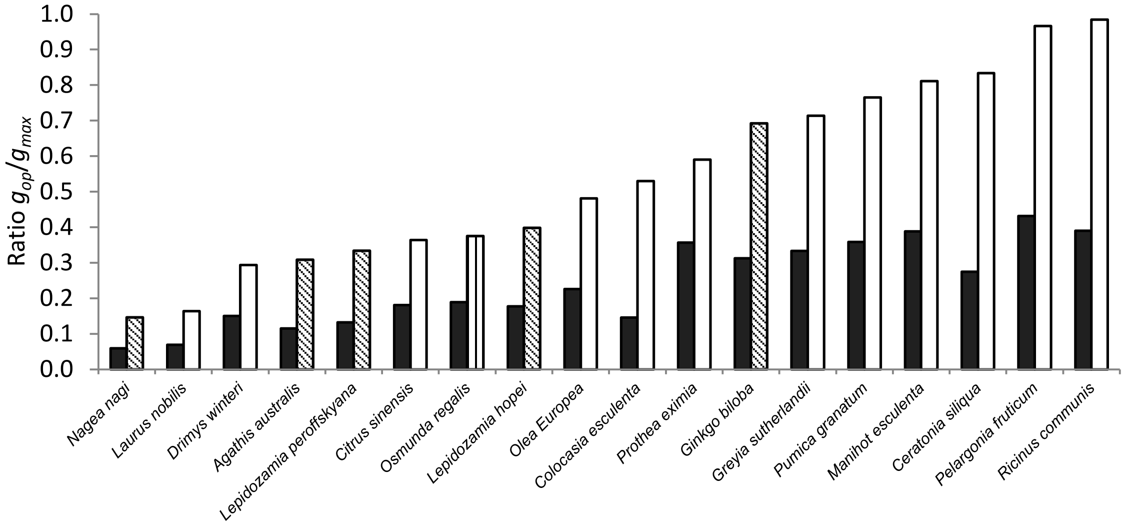


**Fig. S2** Range of scaling relationships observed between mean $g_{op}$ and theoretical $g_{max}$ (closed bars) and the highest single recorded $g_{op}$ / $g_{max}$ for gymnosperms (hatched bars), a fern (vertical-striped bars) and angiosperms (open bars).

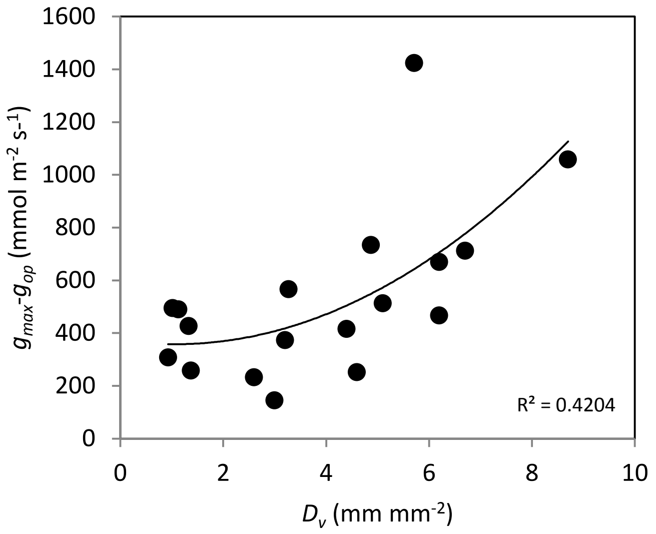


**Fig. S3** Graph showing that disparity between $g_{max}$ and $g_{op}$ increases with increasing $D_v$.

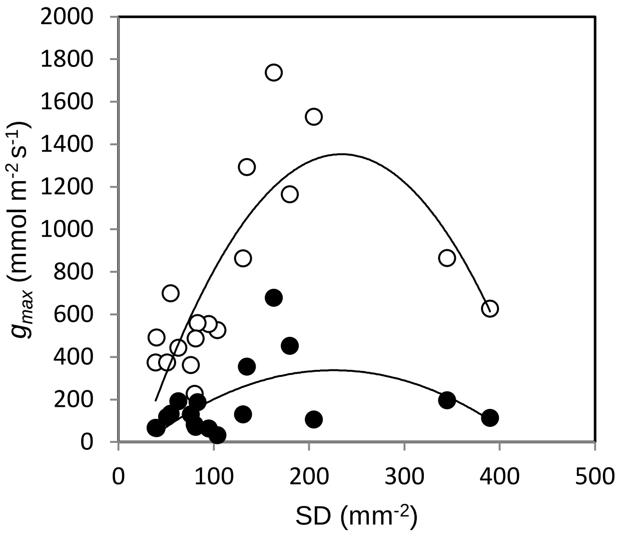


**Fig. S4** Graph illustrating relationship between maximum theoretical stomatal conductance ($g_{max}$) and stomatal density (SD, closed circles: $r^2$ = 0.6675) and between operational stomatal conductance ($g_{op}$) and SD (open circles, $r^2$ = 0.3255).

**Table S1** Estimated stem and crown ages of species lineages studied

| Species | Crown Age (Ma.) | Stem Age (Ma.) | Reference (crown age) | Reference (stem age) |
|---|---|---|---|---|
| ***Lepidozamia hopei*** | 6–15 (Lepidozamia) | 280–300 (Cycadales) | Nagalingum *et al.* (2011); Salas-Leiva *et al.* (2013) | Nagalingum *et al.* (2011); Salas-Leiva *et al.* (2013) |
| ***Nageia nagi*** | 65–23 (Nageia) | 177–237 (Podocarpales) | Biffin *et al.* (2012) | Biffin *et al.* (2012) |
| ***Agathis australis*** | 65–23 (Agathis) | 177–237 (Araucariales) | Biffin *et al.* (2010) | Biffin *et al.* (2010) |
| ***Lepidozamia peroffskyana*** | 6–15 (Lepidozamia) | 280–300 (Cyacdales) | Nagalingum *et al.* (2011); Salas-Leiva *et al.* (2013) | Nagalingum *et al.* (2011); Salas-Leiva *et al.* (2013) |
| ***Ginkgo biloba*** | 146–100 (Ginkgo) | 260 (Ginkgoales) | Crane (2013) | Crisp & Cook (2011) |
| ***Osmunda regalis*** | 66–100 (Osmunda) | 364 (Osmundales) | Pryer *et al.* (2004); Schuettpelz & Pryer (2009) | Yatabe *et al.* (1999) |
| ***Drimys winteri*** | *c.* 21 (Drimys) | 128 (Canellelales) | Müller *et al.* (2015) | Forest & Chase (2009) |
| ***Protea eximia*** | *c.* 35 (Protea) | 123 (Proteales) | Magallon & Castillo (2009); Sauquet *et al.* (2009) | Magallon & Castillo (2009) |
| ***Punica granatum*** | *c.* 35 (Lythraceae) | 108 (Myrtales) | Sytsma *et al.* (2004) | Magallon & Castillo (2009) |
| ***Greyia sutherlandii*** | *c.* 25(Greyia) | 108 (Geraniales) | Sytsma *et al.*, (2014) | Magallon & Castillo (2009) |
| ***Laurus nobilis*** | *c.* 80 (Lauraceae) | 129 (Laurales) | Magallon & Castillo (2009) | Magallon & Castillo (2009) |
| ***Colocasia esculenta*** | *c.* 23 (Colocasia) | 126–138 (Alismatales) | Nauheimer *et al.* (2012) | Anderson & Janssen (2009) |
| ***Pelargonum 'robert fish'*** | *c.* 25 (Pelargonum) | 108 (Geraniales) | Sytsma *et al.* (2014) | Magallon & Castillo (2009) |
| ***Citrus sinensis*** | 12–28 (Aurantioideae) | 98 (Sapindales) | Pfeil & Crisp (2008) | Magallon & Castillo (2009) |
| ***Ceratonia siliqua*** | *c.* 39–59 (Caesalpinoideae) | 101 (Fabales) | Lavin *et al.* (2005) | Magallon & Castillo (2009) |
| ***Olea Europea*** | 23–30 (Olea) | 79 (Lamiales) | Besnard *et al.* (2009) | Magallon & Castillo (2009) |
| ***Manihot esculenta*** | 6.6 (Manihot) | 98 (Malpighiales) | Chacón *et al.* (2008) | Magallon & Castillo (2009) |
| ***Ricinus communis*** | 41? (Ricinus) | 98 (Malpighiales) | Reineke *et al.* (2011) | Magallon & Castillo (2009) |

Ma., million yr ago.

**Table S2** Species investigated and number of replicates in repeat analysis dataset October 2015 $g_{op(\mathrm{max})}$

| Species | Number of measurements | Number of individuals | Leaves per individual |
|---|---|---|---|
| *Agathis australis* | 10 | 3 | 3–4 |
| *Ginkgo biloba* | 14 | 14 | 1 |
| *Lepidozamia hopei* | 10 | 10 | 1 |
| *Lepidozamia peroffskyana* | 11 | 11 | 1 |
| *Nageia nagi* | 10 | 10 | 1 |
| *Osmunda regalis* | 10 | 10 | 1 |
| *Ceratonia siliqua* | 10 | 1 | 10 |
| *Citrus sinensis* | 6 | 1 | 6 |
| *Colocasia esculenta* | 12 | 4 | 3 |
| *Drimys winteri* | 10 | 4 | 2–3 |
| *Greyia sutherlandii* | 10 | 3 | 3–4 |
| *Laurus nobilis* | 10 | 10 | 1 |
| *Manihot esculenta* | 9 | 1 | 9 |
| *Olea europaea* | 10 | 2 | 5 |
| *Pelargonium fruticosum* | 9 | 3 | 3 |
| *Protea eximia* | 9 | 3 | 3 |
| *Punica granatum* | 10 | 1 | 10 |
| *Ricinus communis* | 14 | 2 | 7 |

**References**


**Anderson CL, Janssen T. 2009.** Monocots. In: Hedges SB, Kumar S, eds. *Timetree of life*. New York, NY, USA: Oxford University Press, 203–212.

**Besnard G, de Casas RR, Christin P-A, Vargas P. 2009.** Phylogenetics of *Olea* (Oleaceae) based on plastid and nuclear ribosomal DNA sequences: tertiary climatic shifts and lineage differentiation times. *Annals of Botany* **104:** 143–160.

**Biffin E, Brodribb TJ, Hill RS, Thomas P, Lowe AJ. 2012.** Leaf evolution in Southern Hemisphere conifers tracks the angiosperm ecological radiation. *Proceedings of the Royal Society B: Biological Sciences* **279:** 341–348.

**Biffin E, Hill RS, Lowe AJ. 2010.** Did kauri (*Agathis*: Araucariaceae) really survive the Oligocene drowning of New Zealand? *Systematic Biology* **59:** 594–602.

**Chacón J, Madriñán S, Debouck D, Rodriguez F, Tohme J. 2008.** Phylogenetic patterns in the genus *Manihot* (Euphorbiaceae) inferred from analyses of nuclear and chloroplast DNA regions. *Molecular phylogenetics and evolution* **49:** 260–267.

**Crane PR. 2013.** Ginkgo*: the tree that time forgot*. New Haven, CT, USA: Yale University Press.

**Crisp MD, Cook LG. 2011.** Cenozoic extinctions account for the low diversity of extant gymnosperms compared with angiosperms. *New Phytologist* **192:** 997–1009.

**Forest F, Chase MW. 2009.** *Eurosid I*. In: Hedges SB, Kumar S, eds. *Timetree of life*. New York, NY, USA: Oxford University Press, 188–197.

**Lavin M, Herendeen PS, Wojciechowski MF. 2005.** Evolutionary rates analysis of Leguminosae implicates a rapid diversification of lineages during the Tertiary. *Systematic Biology* **54:** 575–594.

**Magallon S, Castillo A. 2009.** Angiosperm diversification through time. *American Journal of Botany* **96:** 349–365.

**Müller S, Salomo K, Salazar J, Naumann J, Jaramillo A, Neinhuis C, Feild TS, Wanke S. 2015.** Intercontinental long-distance dispersal of Canellaceae from the New-to the Old World revealed by a nuclear single copy gene and chloroplast loci. *Molecular Phylogenetics and Evolution* **84:** 205–219.

**Nagalingum N, Marshall C, Quental T, Rai H, Little D, Mathews S. 2011.** Recent synchronous radiation of a living fossil. *Science* **334:** 796–799.

**Nauheimer L, Metzler D, Renner SS. 2012.** Global history of the ancient monocot family Araceae inferred with models accounting for past continental positions and previous ranges based on fossils. *New Phytologist* **195:** 938–950.

**Pfeil BE, Crisp MD. 2008.** The age and biogeography of *Citrus* and the orange subfamily (Rutaceae: Aurantioideae) in Australasia and New Caledonia. *American Journal of Botany* **95:** 1621–1631.

**Pryer KM, Schuettpelz E, Wolf PG, Schneider H, Smith AR, Cranfill R. 2004.** Phylogeny and evolution of ferns (monilophytes) with a focus on the early leptosporangiate divergences. *American Journal of Botany* **91:** 1582–1598.

**Reineke AR, Bornberg-Bauer E, Gu J. 2011.** Evolutionary divergence and limits of conserved non-coding sequence detection in plant genomes. *Nucleic Acids Research* **39:** 6029–6043.

**Salas-Leiva DE, Meerow AW, Calonje M, Griffith MP, Francisco-Ortega J, Nakamura K, Stevenson DW, Lewis CE, Namoff S. 2013.** Phylogeny of the cycads based on multiple single-copy nuclear genes: congruence of concatenated parsimony, likelihood and species tree inference methods. *Annals of Botany* **112:** 1263–1278.

**Sauquet H, Weston PH, Anderson CL, Barker NP, Cantrill DJ, Mast AR, Savolainen V. 2009.** Contrasted patterns of hyperdiversification in Mediterranean hotspots. *Proceedings of the National Academy of Sciences, USA* **106:** 221–225.

**Schuettpelz E, Pryer KM. 2009.** Evidence for a Cenozoic radiation of ferns in an angiosperm-dominated canopy. *Proceedings of the National Academy of Sciences, USA* **106:** 11200–11205.

**Sytsma KJ, Litt A, Zjhra ML, Pires JC, Nepokroeff M, Conti E, Walker J, Wilson PG. 2004.** Clades, clocks, and continents: historical and biogeographical analysis of Myrtaceae, Vochysiaceae, and relatives in the southern hemisphere. *International Journal of Plant Sciences* **165:** S85–S105.

**Sytsma KJ, Spalink D, Berger B. 2014.** Calibrated chronograms, fossils, outgroup relationships, and root priors: re-examining the historical biogeography of Geraniales. *Biological Journal of the Linnean Society* **113:** 29–49.

**Yatabe Y, Nishida H, Murakami N. 1999.** Phylogeny of Osmundaceae inferred from rbcL nucleotide sequences and comparison to the fossil evidences. *Journal of Plant Research* **112:** 397–404.
